# Supplementary material for: Dual Responsive Dependent Background Color Based on Thermochromic 1D Photonic Crystal Multilayer Films
Source: Polymers (Basel). 2022 Dec 6;14(23):5330. doi: 10.3390/polym14235330 (PMC9735666; doi:10.3390/polym14235330)
Supplement: Supplementary file 1 [file polymers-14-05330-s001.zip › polymers-2083605-supplementary.pdf]

Dual responsive dependent background color based on thermochromic 1D photonic crystal multilayer films

Yejin Kim, Seo Hyun Kim, Henok Getachew Girma, Seungju Jeon, Bogyu Lim and Seo-Hyun Jung**

Center for Advanced Specialty Chemicals, Korea Research Institute of Chemical Technology (KRICT), Ulsan, 44412, Republic of Korea

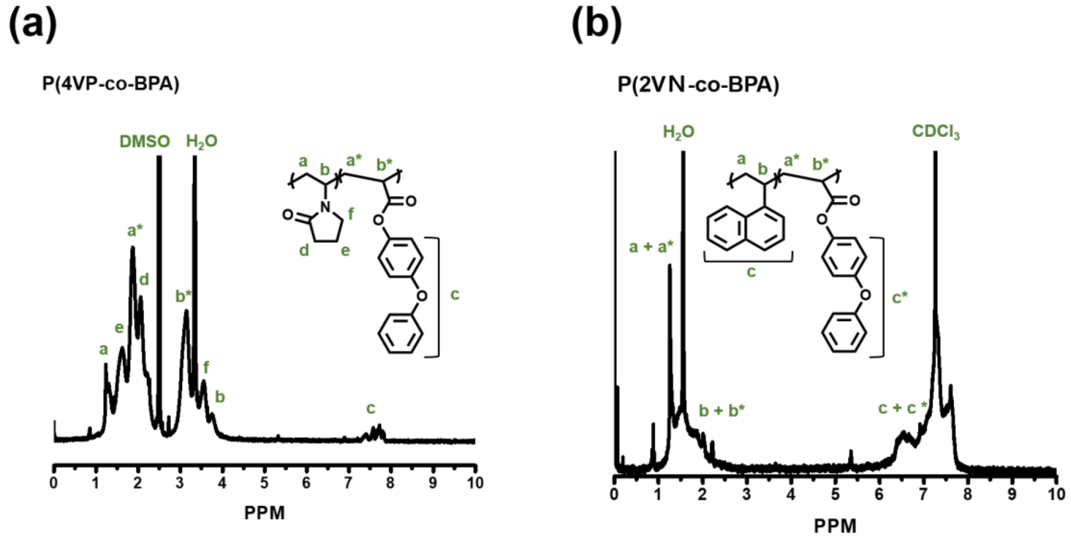

Figure S1. ^{1}H NMR spectra of (a) P(4VP-co-BPA) and (b) P(2VN-co-BPA)

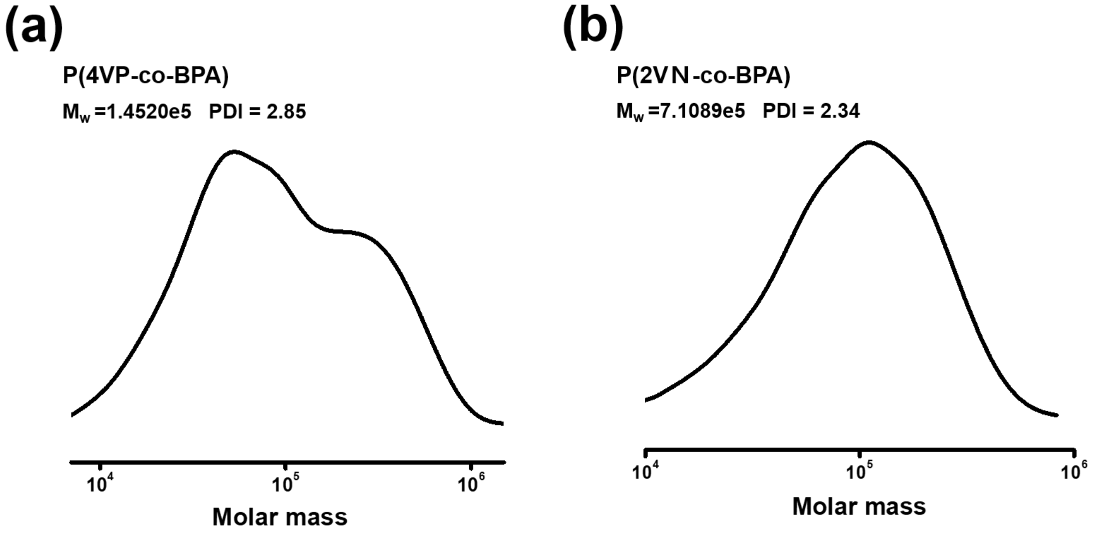

Figure S2. GPC traces of (a) P(4VP-co-BPA) and (b) P(2VN-co-BPA)
